# High-resolution dynamic imaging and quantitative analysis of lung cancer xenografts in nude mice using clinical PET/CT

**DOI:** 10.18632/oncotarget.17263

**Published:** 2017-04-20

**Authors:** Ying Yi Wang, Kai Wang, Zuo Yu Xu, Yan Song, Chu Nan Wang, Chong Qing Zhang, Xi Lin Sun, Bao Zhong Shen

**Affiliations:** ^1^ TOF-PET/CT/MR center, the Fourth Hospital of Harbin Medical University, Heilongjiang, China; ^2^ Molecular Imaging Research Center, Harbin Medical University, Heilongjiang, China; ^3^ Molecular Imaging Program at Stanford (MIPS), Department of Radiology, Stanford University School of Medicine, Stanford, California, USA

**Keywords:** PET/ CT, dynamic scan, molecular imaging, lung cancer

## Abstract

Considering the general application of dedicated small-animal positron emission tomography/computed tomography is limited, an acceptable alternative in many situations might be clinical PET/CT. To estimate the feasibility of using clinical PET/CT with [F-18]-fluoro-2-deoxy-D-glucose for high-resolution dynamic imaging and quantitative analysis of cancer xenografts in nude mice. Dynamic clinical PET/CT scans were performed on xenografts for 60 min after injection with [F-18]-fluoro-2-deoxy-D-glucose. Scans were reconstructed with or without SharpIR method in two phases. And mice were sacrificed to extracting major organs and tumors, using *ex vivo* γ-counting as a reference. Strikingly, we observed that the image quality and the correlation between the all quantitive data from clinical PET/CT and the *ex vivo* counting was better with the SharpIR reconstructions than without. Our data demonstrate that clinical PET/CT scanner with SharpIR reconstruction is a valuable tool for imaging small animals in preclinical cancer research, offering dynamic imaging parameters, good image quality and accurate data quatification.

## INTRODUCTION

PET/CT (positron emission computed tomography combination with X-computed tomography) is a powerful tool for tumor diagnosis, prognosis and relavent research in clinical. With specific radiotracers, it can reflect organs physiological, pathological, biochemical, metabolic and even tumor marker changes at the molecular level. Meanwhile, CT offers high anatomical resolution and provides attenuation correction for PET images [1, 2]. Nowadays, to satisfy the requirements of preclinical cancer rearch programmes, dedicated small-animal PET/CT scanners have been developed to image tumor animal models for *in vivo* studying the cancer at the molecular level, or to exploit more novel PET/CT radiotracers. Small-animal PET/CT scanners are constructed specifically, high-performing systems highly adapted to the morphology and physiology of small animals [3]. It plays an important role as a bridge between the mechanisms of basic disease research and clinical medical transformation.

However, such dedicated small-animal PET/CT scanners are very expensive and have high maintenance costs, in addition, the relatively long imaging time and the single application functionality that are unsustainable for most clinical medical institutions. These preclinical scanners are therefore less widely available than clinical PET/CT imaging and are currently unable to meet the demand for the use. So many researchers have invested efforts into using clinical PET/CT to its full potential in preclincal studies [4], for example, Karine et al. [5] had studied the ability to use clinical PET/CT to detect and investigate head and neck cancers, the smallest lesion detected and measured 3 × 3 × 4 mm, they demonstrated that clinical PET/CT is a feasible examination to detect primary tumors in small animal models, but they used chemically induced tumors which leaded to spend a lot of time and the rate of establishing tumor model successfully was low; Nicolas et al. [6]evaluated the state-of-the-art clinical PET/CT technology in performing static and dynamic imaging of several mice simultaneously, but they didn't study the metabolism in tumor performed on dynamic imaging. And both didn't compare a clinical PET/CT with a dedicated small animal PET.

Notably, while the performance of clinical PET/CT can't match with dedicated small animal scanners due to limited spatial resolution, both share the same principles and functions. Furthermore, with the continuous improvement of traditional detectors and new technologies such as SharpIR steadily emerging, the image quality and the accuracy of quantitative analyses in clinical PET/CT can be significantly improved theoretically [7, 8]. According to the manufacturers, SharpIR is based on the detector response of the PET scanner in the iterative system model. The application incorporates information about the PET detector response into the 3D iterative reconstruction algorithm, which forms a new advanced reconstruction algorithm based on the point spread function. It can enhance visual contrast and resolution in PET images and also can improve the accuracy of underlying model. Because lung cancer is susceptible to respiration during PET/CT imaging, so it's difficult to gain high-quality PET/CT image of small animals. But with the emergence of the advanced reconstruction techniques, there is a significant advantage in improving breathing movement, which is beneficial to lung cancer research. In the study, our purpose were: a. To explore the feasibility of clinical PET/CT dynamic imaging in tumor-bearing mouse model and how to optimize the scan parameters and new reconstruction algorithm; b. To investigate the viability of using clinical PET/CT scanner to gain high-quality image, especially with advanced SharpIR reconstruction technique; c. To explore the capability of accurate quantifying radioactivity concentration noninvasively of main organs *in vivo*.

## RESULTS

### ^18^F-FDG PET/CT images

**Figure 1:**
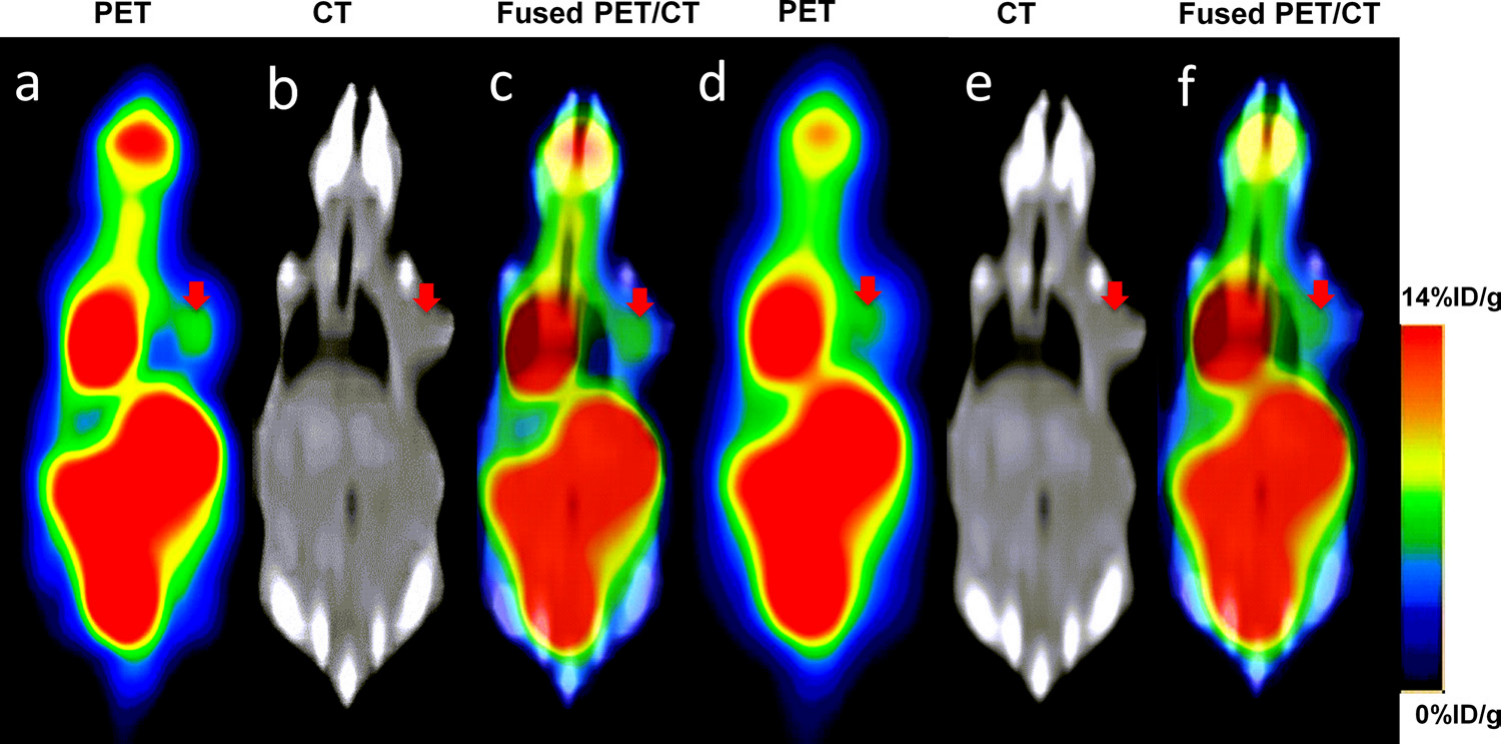
Coronal slices of a 23g mouse bearing subcutaneous tumour injected with ^18^F-FDG for clinical PET/CT imaging Increased ^18^F-FDG uptake was readily observed on the right shoulder of the mouse on PET, CT and fused PET/CT images. **(a∼c)** reconstruction with SharpIR; **(d∼f)** reconstruction without SharpIR. PET slices have been scaled to the same maximum and all images were the same slice. The red arrow represents the tumor region.

^18^F-FDG PET/CT images clearly revealed H1975 (non-small cell lung cancer cell line) lung xenografts growing subcutaneously in the nude mice (Figure 1a∼1c). High ^18^F-FDG uptake was detected in the tumor on the right shoulder in coronal PET images, and this could be confirmed in coronal CT images. We could see increased uptake of ^18^F-FDG in the tumor on PET images, but the intratumoral tracer uptake heterogeneity could not be adequately determined using the clinical PET/CT scanner, probably because the tumors were small in size. The reconstructions with SharpIR (Figure 1a∼1c) were significantly sharper and clearer than those without SharpIR (Figure 1d∼1f). Organs such as brain, kidneys, bladder, heart and intestines showed high ^18^F-FDG uptake. To evaluate the clinical PET/CT image quality, we also used SNR (Signal to Noise Ratio) and scores attributed independently by three experienced nuclear medicine doctors (Table 1). Both SNR and scores clearly demonstrate that clinical PET/CT images reconstructed with SharpIR (SNR = 0.449 ± 0.09; 5.56 ± 0.58 scores) had significantly higher quality than reconstructed without SharpIR (SNR = 0.173 ± 0.09; 3.78 ± 0.86 scores, *P* < 0.01). Paired Student's t-test was used to assess these differences.

**Table 1:** Comparison of SNR and scores from clinical PET/CT reconstucted with and without SharpIR

Groups	No. of mice	SNR	Scores
**With SharpIR**	10	0.449 ± 0.09**	5.56 ± 0.58**
**Without SharpIR**	10	0.173 ± 0.09	3.78 ± 0.86

Abbreviation: SNR, signal to noise ratio. Paired Student's t-test was used to evaluate the differences. **, *P* < 0.01.

### Quantification capability of clinical PET/CT scanners for small animal imaging

**Figure 2:**
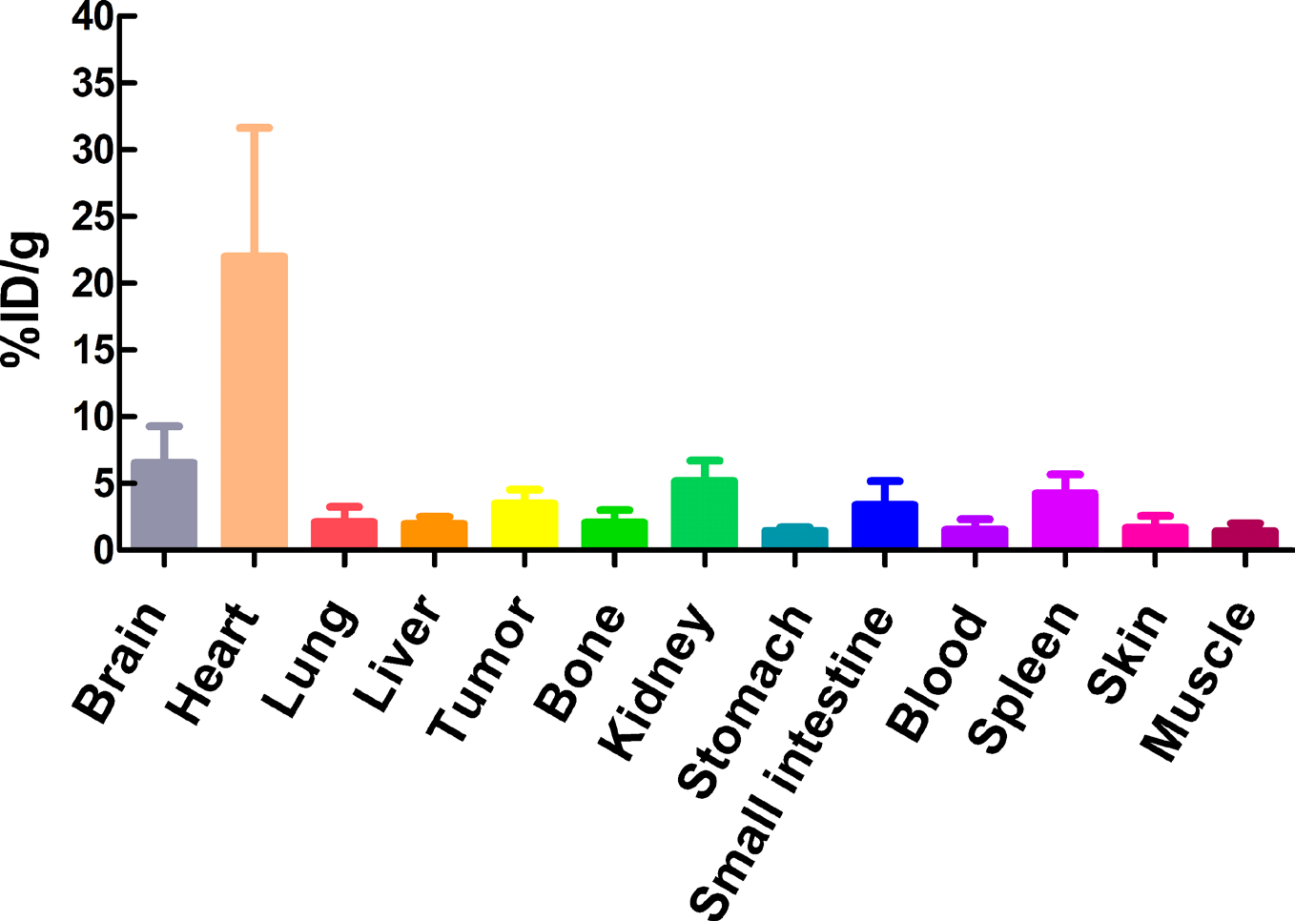
The biodistribution of some organs and tumors in mice The γ-counter quantification analysis for uptakes of some organs and tumors for ^18^F-FDG in H1975 xenograft models (n=10) after dynamic imaging 60min.

**Figure 3:**
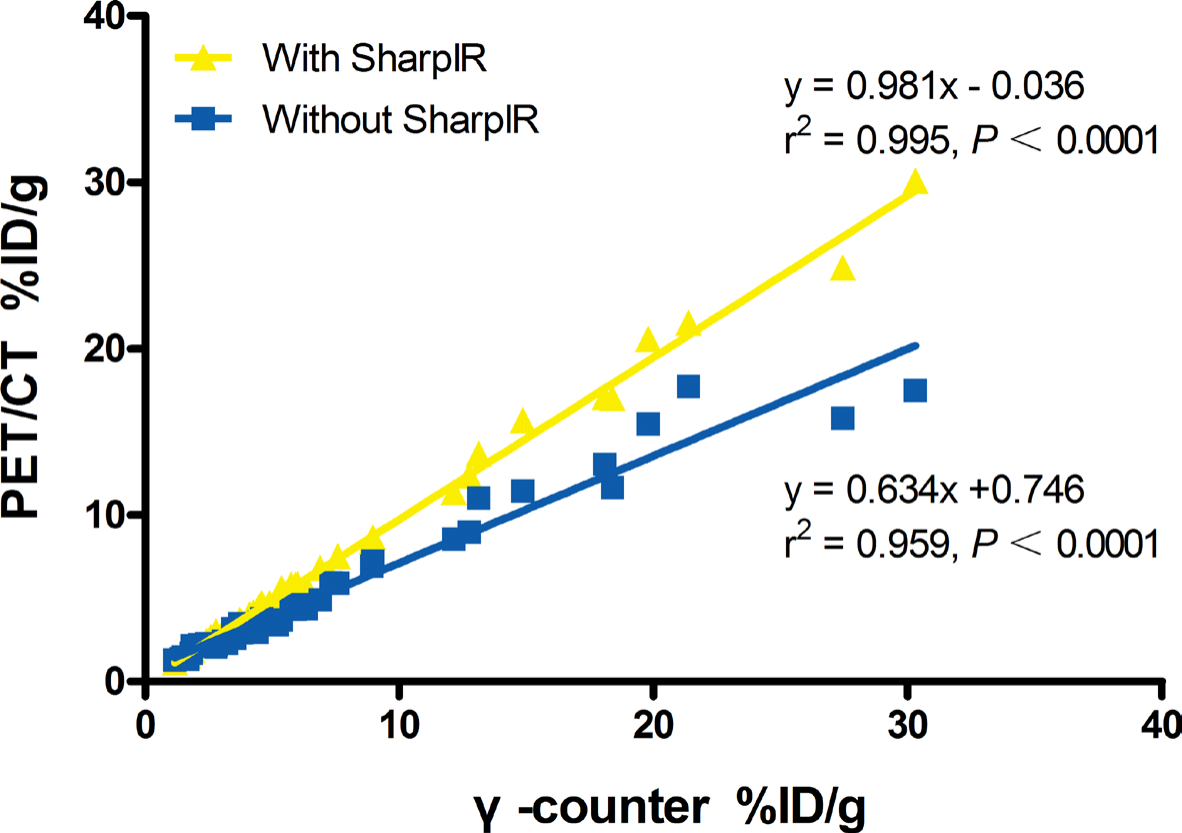
Linear regression analysis of %ID/g from clinical PET/CT versus γ-counter The %ID/g of main tissues extracted from PET/CT with SharpIR reconstruction and that by *ex vivo* counting has a strong correlation (r^2^ = 0.995). A marked improvement of the slope (0.981) when data was reconstructed with SharpIR reconstruction algorithm compared to without SharpIR (0.634).

The %ID/g of main tissues (brain, heart, liver, kidney, tumor and muscle) from clinical PET/CT images reconstucted with SharpIR was higher than that without SharpIR (Table 2), the former was more similar to the quantification on γ-counter. The biodistribution histogram for tumor and several organs (Figure 2) shows that the metabolic distribution of ^18^F-FDG activity was consistent with the PET/CT images (Figure 1); the most prominent was in the normal heart (22.01 ± 9.64), followed by brain (6.54 ± 2.73) and kidney (5.18 ± 1.55), with low ^18^F-FDG uptake in other organs. In tissues, there was a high compliance between the radioactivity concentrations estimated from clinical PET images and those directly measured by γ-counting in normal organs and tumors (both were transformed into %ID/g). The correlation of %ID/g obtained from clinical PET/CT with SharpIR reconstruction (r^2^ = 0.995, slope = 0.981, *P* < 0.0001) was better than without (r^2^ = 0.959, slope = 0.634, *P* < 0.0001) both compared to that from *ex vivo* counting (Figure 3).

**Table 2:** Comparison of %ID/g and SUVmax calculated by ^18^F-FDG PET/CT vs *ex vivo* γ-counter

Organs	%ID/g on γ-counter	%ID/g on clinical PET/CT(with SharpIR)	%ID/g on clinical PET/CT(without SharpIR)	%ID/g on micro-PET/CT	SUV_max_ on clinical PET/CT(with SharpIR)
**Brain**	6.54 ± 2.73	6.23 ± 2.55	5.02 ± 1.99	6.33 ± 1.38	1.19 ± 0.41
**Heart**	22.01± 9.64	21.67 ± 9.42	15.09 ± 5.47	21.19 ± 4.05	3.06 ± 0.96
**Liver**	1.97 ± 0.54	1.93 ± 0.49	1.86 ± 0.43	1.88 ± 0.34	1.09 ± 0.42
**Kidney**	5.18 ± 1.55	5.03 ± 1.54	3.91 ± 1.11	5.10 ± 1.46	1.81 ± 0.45
**Tumor**	3.53 ± 1.02	3.47 ± 0.88	2.76 ± 0.69	3.32 ± 1.09	0.54 ± 0.12
**Muscle**	1.06 ± 0.45	1.01 ± 0.33	0.54 ± 0.19	1.03 ± 0.13	0.22 ± 0.10

Abbreviation: %ID/g, percent intensity dose per gram; SUVmax, maximum standard uptake value. Data are mean ± SD. %ID/g on PET/CT was calculated from the average of two reconstruction mode of each pixel's activity value within each ROI, which totally surrounded the FDG uptake in target tissues. SUVmax was calculated from PET/CT images reconstructed with SharpIR.

**Figure 4:**
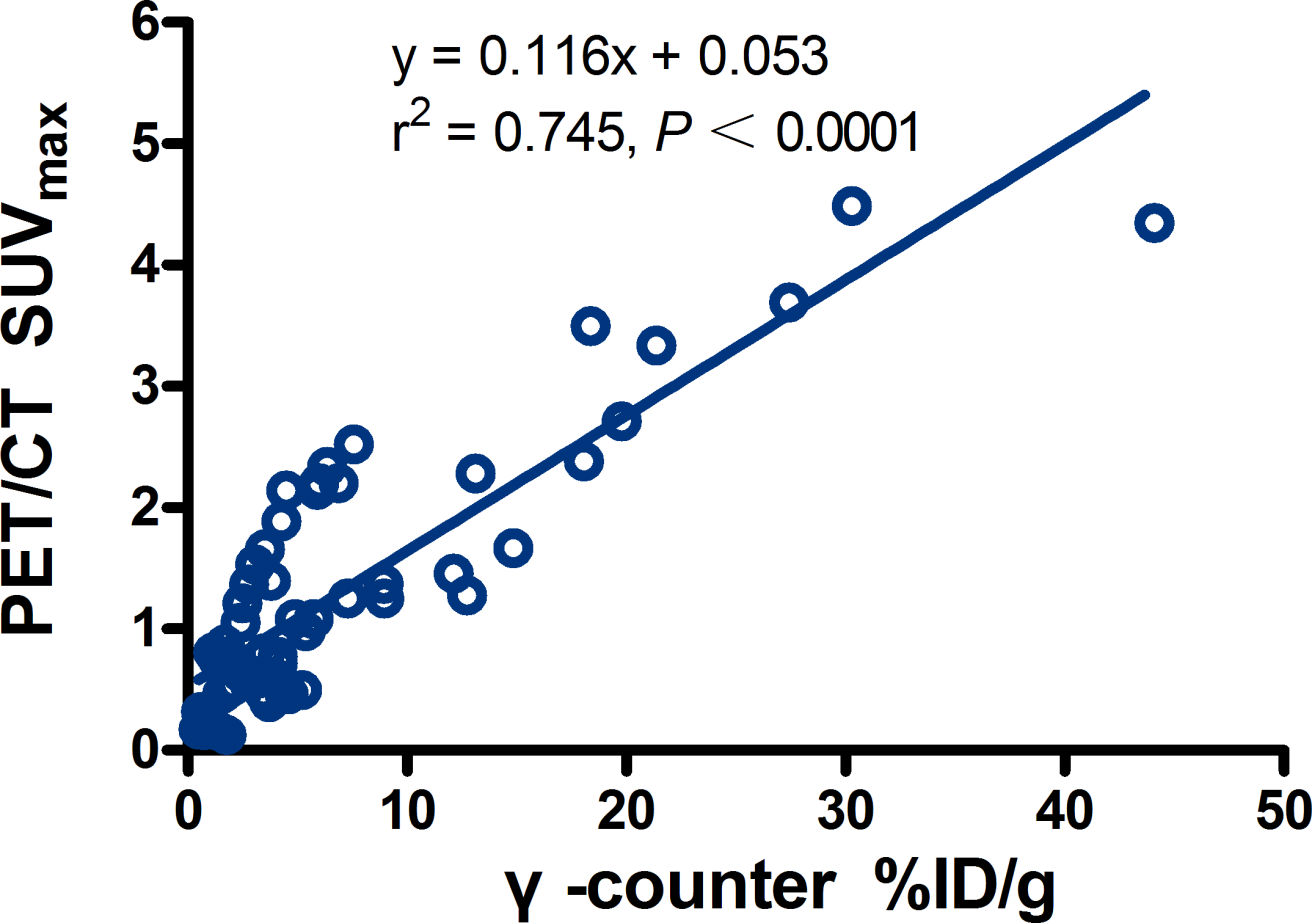
Regression plots for PET/CT SUV_max_ versus %ID/g from γ-counter in main tissues There was a low correlation (r^2^ = 0.745, slope = 0.116; *P* <0.0001).

Although there was also a linear correlation between %ID/g measured from γ-counter and SUV_max_ quantified from clinical PET/CT images with SharpIR reconstruction (r^2^ = 0.745, slope = 0.116, *P* < 0.0001) (Figure 4), it was clearly weaker than %ID/g obtained from PET/CT quantifications (Figure 3). Furthermore, ^18^F-FDG activity in tissues quantified by SUV_max_ was most prominent in the normal heart (3.06 ± 0.96), followed by kidneys (1.81 ± 0.45), brain (1.19 ± 0.41) and liver (1.09 ± 0.42), with very weak uptake in other organs (Table 2).

The T/NT of %ID/g calculated from clinical PET/CT images (3.58 ± 0.73) was similar to that from γ-counter (3.76 ± 1.66), and both were significantly higher than the ^18^F-FDG radioactivity quantified in SUV_max_ on PET/CT images (2.77 ± 0.79, *P* <0.05) (Table 3).

**Table 3:** Comparising the ratio T/NT from PET/CT and γ-counter

Groups	No. of mice	T/NT
**γ-counter(%ID/g)**	10	3.76 ± 1.66
**PET/CT(%ID/g)**	10	3.58 ± 0.73
**PET/CT(SUV_max_)**	10	2.77 ± 0.79*

Abbreviation: T/NT, the ratio of the activity in tumor divided by that in normal tissue. Data are mean ± SD. *, *P* < 0.05.

**Figure 5:**
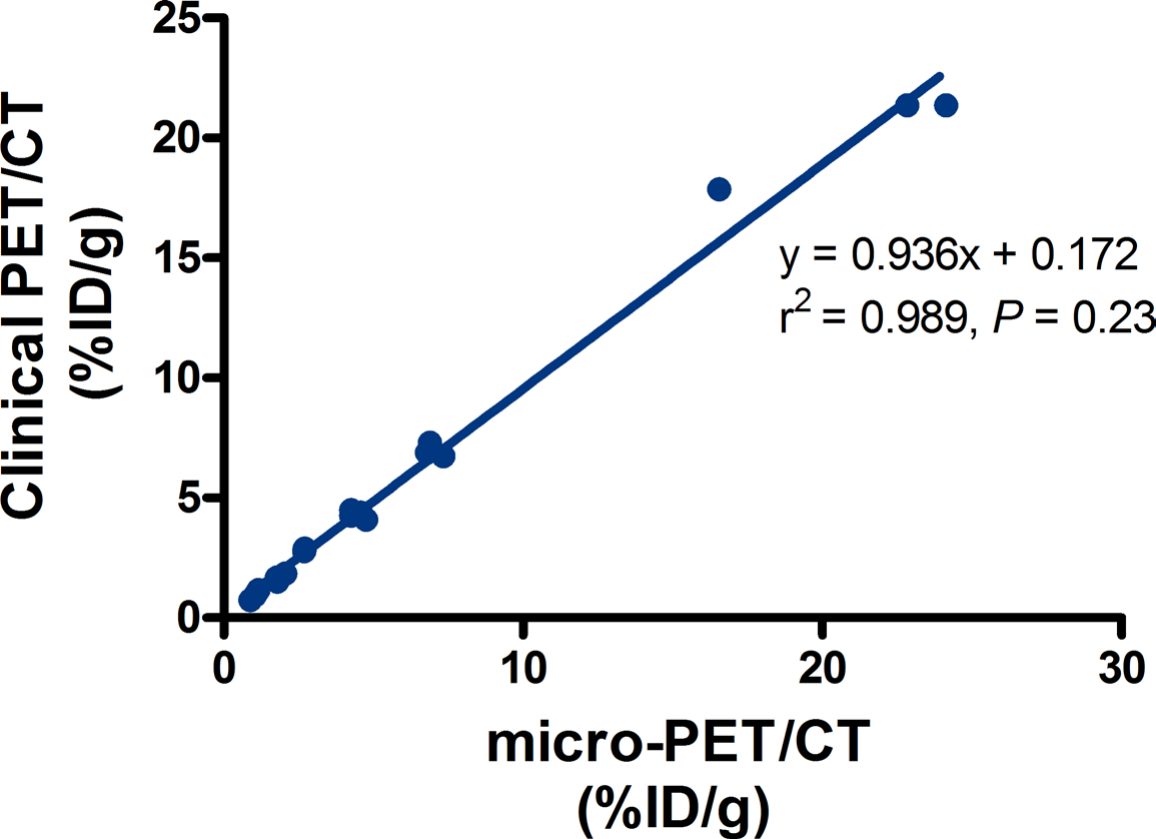
The relationship between the radioactivity uptake %ID/g obtained from clinical PET and micro-PET Data are shown as mean ± SD (*n* = 4).

For comparison of quantification on clinical PET/CT vs micro-PET/CT in small animal imaging, ^18^F-FDG micro-PET/CT confirmed that the %ID/g of tumors and main organs was consistent with the %ID/g quantifications on clinical PET/CT (Table 2). There was a strong correlation between them (r^2^ = 0.989, slope = 0.936), and had no significant difference (*P* = 0.23) (Figure 5).

### Time activity curves and kinetics of FDG uptake

**Figure 6:**
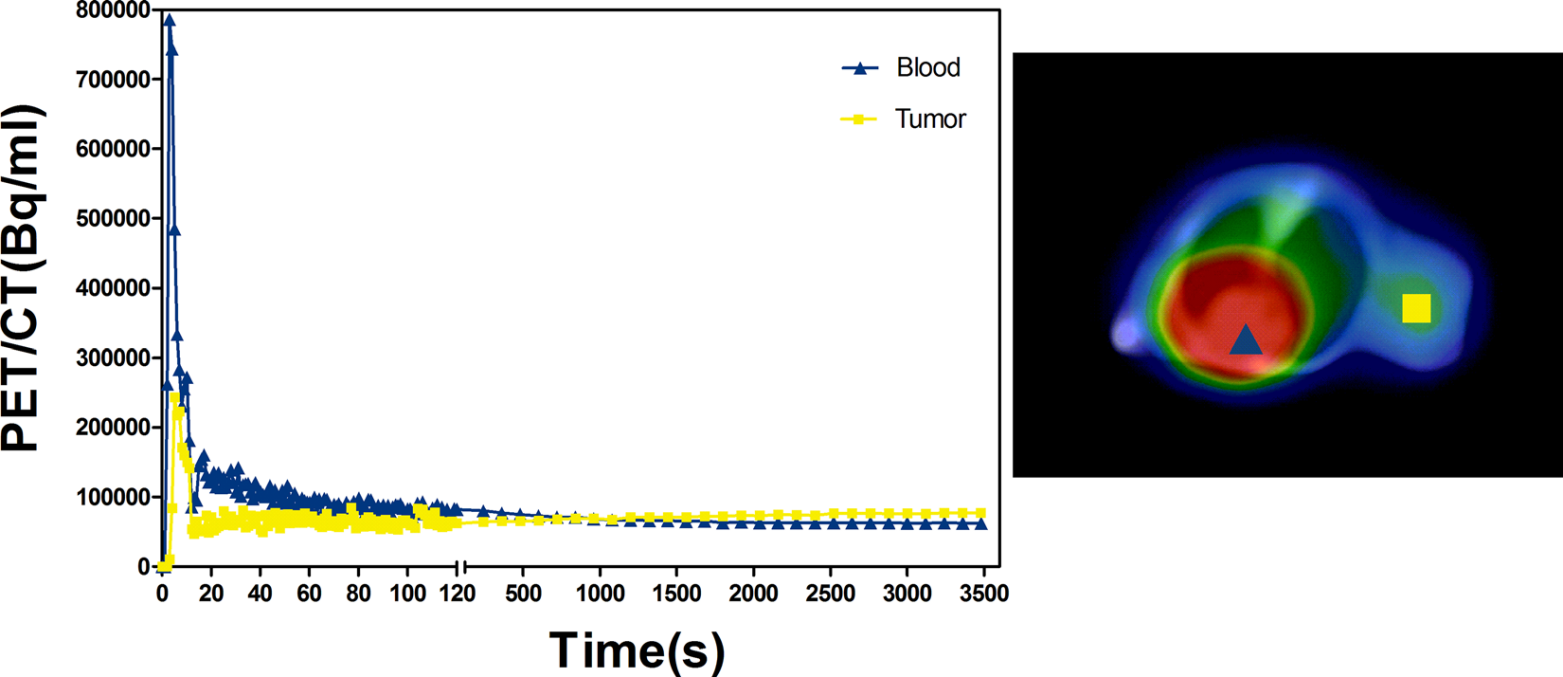
Time–activity curves (TAC) of tumor ROI and blood ROI On fused clinical PET/CT images with SharpIR recounstructed, we marked out the ROIs of tumor and blood. The tumor and blood TACs were explored, the front portion of X axis shows 0-120s, the latter shows 120s-3600s. The triangle represents blood, the square represents tumor.

Typical examples of time activity curves (TACs) extracted from dynamic PET/CT data reconstructed with and without SharpIR were obtained from ROIs drawn over the heart and tumor in each mouse (Figure 6). From the merged PET/CT images, the TACs of tumor ROI and blood ROI were analyzed, with one end of the X axis showing 0-120s and the other showing 120s-3600s. The tumor curve showed fast influx during the early phase and then maintained a steady state. However, the blood curve peaked and dropped quickly, then kept falling slowly; but the tumor time-activity curve peaked and dropped quickly, then continued to rise slowly. We used a standard one-tissue compartment model to simulate the metabolism of the tumor region, with parameters including regional uptake K_1_ (mL/min/g) and clearance K_2_ (min^−1^). Paired student t-test was used to assess the differences in K_i_ between reconstruction with and without SharpIR. Both regional uptake parameters K_1_ and clearance parameters K_2_ were higher after post-processing with SharpIR (K_1_ = 0.23 ± 0.13; K_2_ = 0.0044 ± 0.0015) than without SharpIR (K_1_ = 0.08 ± 0.03; K_2_ = 0.0023 ± 0.0009, *P* < 0.01) (Table 4).

**Table 4:** Comparison of K_1_ and K_2_ with and without SharpIR on clinical PET/CT

Groups	No. of mice	K_1_(mL/min/g)	K_2_(min^−1^)
**With SharpIR**	10	0.23±0.13**	0.0044±0.0015**
**Without SharpIR**	10	0.08±0.03	0.0023±0.0009

The table showed the value of K_1_ and K_2_ from mice (n=10) with or without reconstruction of SharpIR. Paired Student's t-test was used to evaluate the differences of K_1_ and K_2_ between reconstruction with SharpIR and without SharpIR. **, *P* < 0.01.

## DISCUSSION

Small animal imaging is a powerful and convenient method to assess metabolic states in real-time and, in recent years, it has been increasing used in *in vivo* preclinical studies in various fields including molecular biology, oncology and neuroscience research [9]. PET imaging can provide metabolic and functional information noninvasively, quantitatively and repeatedly. PET's unique feature of reproducing *in vivo* physiological processes in real-time relies on injected positron-labeled specific molecular tracers that track local ROIs metabolic processes, thereby producing so-called time activity curves (TACs). TACs provide kinetic parameters that allow the assessement of physiological processes in space and time. Notably, combined PET/CT techniques can overcome the limitation of PET in detecting precise anatomical locations, by taking advantage of the detailed morphological information provided by high-resolution CT images. While small animal PET (micro-PET) has raised interest in particular for preclinical biomedical research, a limited number of small animal PET/CT scanners are currently commercially available and their high price and maintenance costs limit their versatility. Thus, many researchers now try to use clinical PET/CT for imaging small animals in preclinical studies.

In this study, we compared the SharpIR reconstruction method in the Discovery 790 Elite clinical PET/CT scanner (General Electrics), which is based on the point spread function (PSF) [10], with the traditional Ordered Subsets Expectation Maximization (OSEM) method [11]. Our results clearly show that the signal to noise ratio and the spatial resolution of the PET images could be effectively improved with SharpIR (Figure 1). A previous study investigating the feasibility of clinical PET/CT for small animal imaging used *ex vitro* counting as a reference standard, however, the instrument utilized was a GE Discovery LS PET scanner, which has a reconstruction resolution of 5∼6mm [12]. Moreover, commercial SharpIR reconstruction was not applied and the three-dimensional acquisition mode was not available at that time. As a result, the PET/CT images of tumors and normal organs in mice were less clear than those in rabbits and rats, mainly due to the small volume of the tumors in mice. Here we demonstrate that it is possible to obtain good quality PET images in small animal tumor models (mice) by increasing the spatial resolution of clinical PET to ∼3.3mm, which can be achieved by aquiring the data in three-dimensional acquisition mode and by using one kind of powerful post-processing software to perform SharpIR reconstructions. Our results show that reconstructions with SharpIR or without SharpIR in the same mice produce significantly different images; structures reconstructed with SharpIR had higher resolution and much less distortion than images reconstructed without SharpIR, and the uptake intensity in the tumors was significantly sharper in the SharpIR images (Figure 1).

Clinical PET/CT is currently the best optional quantitative radionuclide imaging apparatus and it is therefore widely used by researchers for *in vivo* functional imaging, blood perfusion of lesions, quantitative studies of drug metabolism, among others. However, the clinical PET/CT quantitative parameters are often limited. For instance, the most commonly used parameter in clinical work, the maximal standard uptake value (SUV_max_), which is normalized by the injected radioactivity and body mass, presents numerous problems as discussed in detail elsewhere [13, 14]. In previous studies, ^18^F-FDG uptake in tissues of small animals has been quantified using both %ID/g and SUV_max_, but %ID/g was more often used in animal experiments because it allows for direct comparisons with biodistribution studies [15]. These %ID/g values were similar to SUV_max_ data, but the SUV_max_ was amenable to confounding variables described in [14], including body characteristics, lesion size, scanner resolution, tracer pharmacokinetics and glycemia. Our %ID/g quantifications, however, revealed a strong relationship between SharpIR reconstructions on PET images and biodistribution data from γ-counter (r^2^ = 0.995, slope = 0.981). Recontructions without SharpIR showed a weaker correlation with γ-counter quantifications (r^2^ = 0.959, slope = 0.634). Notably, we found a very low correlation (r^2^ = 0.745, slope = 0.116) between the regression plots for PET/CT SUV_max_ normalized with body weight and the %ID/g quantifications from γ-counter in main tissues. Moreover, T/NT ratios extracted from %ID/g quantifications (3.58 ± 0.73) showed a better relationship with γ-counter mesurements (3.76 ± 1.66) than SUV_max_ plots (2.77 ± 0.79, *P* < 0.05). These results demonstrated that the %ID/g quantitative method in PET data reconstruction with SharpIR correlates very well with *ex vivo* distrubution data from γ-counter, suggesting that this method is more accurate than the SUV_max_ parameter for quantitive small animal PET/CT imaging. Consistent with the fast mouse heart-rate (300∼500 beats/min, or about 5 times higher than human heart-rate), the highest %ID/g was obtained in heart.

Since PET provides the possibility of absolute data quantification based on compartment model [16], we explored the dynamic scanning data to take full advantage of PET/CT imaging and gain more quantitative information. We compared the kinetics of ^18^F-FDG in reconstructions with or without SharpIR in whole-tumors ROIs using one-compartment data. We found that the accuracy of the kinetic parameters in reconstructions without SharpIR was degraded, probably due to the loss of resolution and sensitivity (Table 4). When we used the time activity curves (TAC) of blood in heart to acquire an input function from PET/CT images and used tumor TAC to acquire an output function, based on dynamic data sets, we found that the regression-based calculation of parametric images was a fast and robust method to produce a slope and intercepted images, which could then be used to calculate K_1_ and K_2_ for all mice. The slope was related to the transport of ^18^F-FDG [6, 17] and the model parameters were estimated in 60 min. Interestingly, the tumor metabolic rate-constant K_1_, K_2_ of ^18^F-FDG was slightly lower than what had been previously described [18], however, this could be explained by the low temperature of the room during the dynamic clinical PET/CT scanning which could have affected the mice's metabolic rate; moreover, their study was about the tumor of melanoma transplanted into the lung of mice, but in this study we used subcutaneous xenograft model of lung cancer. The main downsides in this study was that lung cancer xenografts in nude mice might have heterogeneous diffusion and non-uniform distribution in tumors, but due to the limitations of spatial resolution in clinical PET/CT, we ignored this factor.

Overall, we found that clinical PET/CT image quality (Figure 1a∼1c) can be tremendously improved by advanced reconstruction methods, although can't match the quality of dedicated small animal PET scanners which produce super-resolution images of approximately 1.5 mm. Moreover, clinical PET/CT can also perform noninvasive preclinical dynamic imaging of tumor-bearing small animals, including accurate radioactivity quantification with %ID/g and kinetic parameters. As dedicated small animal PET/CT systems are expensive and scarcely avaiable, the clinical PET/CT scanner could be a valuable alternative for many preclinical studies.

In summary, our study for nude tumor xenograft models injected with ^18^F-FDG and analyzed on a clinical PET/CT scanner, the image quality was significantly improved after reconstruction with SharpIR. The quantitative analysis method using %ID/g measurements on PET images with SharpIR reconstruction produced more accurate quatatifications than using SUV_max_ parameters. Dynamic data based on a one-compartment model can accurately reproduce the tumors’ regional flow. We conclude that imaging and quantification of dynamic metabolic states can be achieved using a clinical PET/CT scanner and the study also could provide a reference where dedicated small animal PET/CT is not available.

## MATERIALS AND METHODS

### Small animal models

All animal studies were executed in accordance with animal protocols approved by the China Guidelines for Animal Care and Ethic for Animal Experiments. The experimental protocols were approved by the Animal Use and Care Committee of Harbin Medical University. Fourteen 5-6 week age BALB/c female nude mice (∼20 g) were purchased from the Slack Laboratory Animal Center in Shanghai. They were fed in SPF environment and had free access to food and water. All mice bearing H1975 human non-small cell lung cancers (NSCLCs) were established with a subcutaneous tumor on their right shoulder. The H1975 cells were purchased from the Institute of Cell Biology in Shanghai and were cultured in RPMI-1640 (GIBCO, Grand Island, USA) containing 10% heat-inactivated fetal bovine serum (FBS) in a 5% CO_2_ incubator at 37°C [19]. Mice were injected on the right shoulder subcutaneously with 6×10^6^/0.1ml of H1975 cells and were used for imaging studies 3–4 weeks after inoculation when the tumor volume (V = L*W^2^/2, L = length, W = width) about 200 mm^3^.

### *In vivo* PET/CT imaging

Scans were acquired on a clinical time-of-flight (TOF) PET/CT (64-slice spiral computed tomography) scanner (Discovery 790 Elite; GE healthcare). This scanner allows for the simultaneous acquisition of 47 transaxial images with an interslice spacing of 3.3 mm in each bed position. The synthesis of ^18^F-FDG was performed at our center in the GE TracerLab FN_FDG_ cyclotron with radiochemical purity reaching 99%. Before performing PET scanning, we conducted multidetector row helical CT scanning, which was used for attenuation correction and anatomical orientation. The technical parameters selected for CT imaging were: tube voltage of 120kVp; tube current of 100mAs; matrix of 192×192; scan FOV (field of view) of 50cm. The technical parameters selected for PET imaging were: slice thickness of 3.3mm; scan FOV of 50cm.

After CT scanning, a PET phantom scan was performed for calculating the conversion factor (CF) of this clinical PET/CT dual system as follows: the radiopharmaceutical ^18^F-FDG was roughly diluted into 1μCi/ml of purified water, then 1000ml ^18^F-FDG was taken out and placed in a well-type meter to measure the radioactivity and immediately scanned by the PET/CT. The initial dose of ^18^F-FDG, the initial measurement time of the phantom using well-type meter, the scan time, and the weight of the phantom for clinical PET/CT imaging were recorded. The CF was calculated by dividing radioactivity per gram (μCi/g) by radioactivity per milliliter (kBq/ml).

Ten of the mice (18∼22g) fasted for at least 4h before imaging and then anesthetized with 1%∼2% isoflurane inhalation. Each animal was fixed on the scan bed in the prone position with all limbs fully extended. For the dynamic scan, a self-restraint cannula was placed in the tail vein under general anaesthesia. A bolus of ^18^F-FDG tracer (3.7 MBq∼7.4MBq/0.2ml) was injected manually into the tail vein and immediately followed by the clinical PET scan. Dynamic ^18^F-FDG clinical PET scans were performed on each mouse for 60min and the animals were kept in the anesthetized condition from the time of ^18^F-FDG administration until the end of imaging. A whole-body emission clinical PET scan for the same axial coverage was performed in the 3D-mode and the data was stored in list mode, which we could also use to reconstruct the data [20].

To gain a basis for comparing %ID/g from clinical PET/CT, we had imaged with a typical micro-PET/CT scanner (SuperArgue; SEDECAL) in the remaining four small animal models. The four mice had at least 4h fasting before scanning and then were anesthetized with 1%∼2% isoflurane inhalation. A solution of ^18^F-FDG (3.7 MBq ∼7.4MBq/0.2ml) tracer injection was performed via the tail vein. To minimize the uptake of ^18^F-FDG in muscle and brown fat, animals were kept anaesthetized on the heating pad for a 45-min period after injection. Each animal was also fixed on the scan bed in the prone position with all limbs fully extended and scanned 45–60 min after injection.

### Image reconstruction

The dynamic data on clinical PET/CT scanner were reconstructed using two methods: (i) with SharpIR or without SharpIR, reframed as 120 frames × 1s, 29 frames × 2min, total 60min; (ii) with SharpIR or without SharpIR, reframed as 1 frame × 45∼60min, total 15min. The first protocol was scheduled mainly taking into account the input function changing rapidly in the early stage, therefore sampling was very intensive during this phase. All reconstructed frames were transferred to a computer workstation (Xeleris2.0, GE Healthcare). The second protocol was mostly considered as a static scan, and was used for assessing the quality of the images and for quantifying. The reconstructed images including PET, CT and fused PET/CT images, were generated on the advantage workstation version (AW4.6 software package, GE healthcare).

The transaxial full width at half maximum (FWHM) of the microPET is 1.66 mm in close to the center of the field of view. The micro-PET images were reconstructed by classical 3D oedered subsets expectation maximization (OSEM) with 25 iterations.

### Quantitative date acquirement of PET/CT imaging

Two quantitative methods are currently used in preclinical PET/CT studies. The first method is the percentage of the injected dose per gram (%ID/g), which is calculated as the activity in target tissues (calibrated in kBq/mL) divided by the decay corrected activity injected into the animal (given in μCi)×CF (mL/g)×100. In this study, the activity in target tissues obtained from the clinical PET/CT images reconstructed with or without SharpIR 45-60min, and also from micro-PET/CT, both were calculated as %ID/g. 3D regions of interest (ROIs) were placed on transaxial PET images entirely covering the most intense areas of ^18^F-FDG uptake in the brain, heart, liver, kidney, tumor and muscle, while avoiding nearby tissues. The average values (Av) of each pixel's activity within each ROI was calculated (expressed as kBq/mL). As the location of ROIs on the PET images could not be accurately defined, we used the corresponding transaxial CT images as a reference. The decay-corrected radioactivity of average values acquired from ^18^F-FDG PET images was transformed into %ID/g. The detail formula was (1- 4):

%ID/g=(CF×L/I)×100 (1)

$$\text{CF}=B_{1}/B_{2}/B_{3}$$ (2)

$$I=G\times \exp\left(-0.693\times \left(\begin{array}{c} \left(100\times (H-\text{INT}(H))+60\times \text{INT}(H)\right)\\ -(100\times (E-\text{INT}(E))+60\times \text{INT}(E)) \end{array}\right)/109.8\right)$$ (3)

$$G=C\times \exp(-0.693\times \left(\begin{array}{c} \left(100\times (E-\text{INT}(E))+60\times \text{INT}(E)\right)\\ -(100\times (D-\text{INT}(D))+60\times \text{INT}(D)) \end{array}\right)/109.8)-F$$ (4)

B_1_=radioactivity of phantom; B_2_=weight of phantom; B_3_= Average uptake values of phantom from PET images; L= Average values of tissues from PET images; I= scan dose; CF= conversion factor; G= injected dose; H= scan time; E= injected time; D= initial time; C= initial dose; F= residual dose; INT is a function that was rounded down to the nearest integer;

The other method which takes animal weight into account is the standardized uptake value (SUV) calculated as the activity concentration (μCi/ml) in tissue divided by the decay-corrected activity injected into the animal (μCi/g) [21]. The activity of main tissues was obtained from 3D ROIs on attenuation-corrected PET images as described above, and was decay-corrected to time of injection. The obtained value is almost unitless, but in fact its unit is g/ml, which is a semi-quantitative value to measure tissue radioactivity. The details are described elsewhere [22], in the study, we used the maximum SUV (SUV_max_) as quantitative parameter, according to the following formula:

$$\text{SUV}=\frac{\text{TA}\left(\mu \text{Ci}/\text{ml}\right)}{\text{IA}\left(\mu \text{Ci}\right)/W\left(\text{g}\right)}$$ (5)

TA= tissue activity on PET images; IA= injected activity; W= weight of animal.

To assess tumor metabolic activity, the quantification of relative tumor uptake was accomplished by comparing tumor to normal tissue (T/NT), with muscle being considered as normal tissue. The T/NT ratios of %ID/g and SUV_max_ were extracted from the volume of interest (VOIs) on clinical PET images.

To evaluate the quality of PET, CT and fused PET/CT images, the images were reviewed and scored independently by three experienced nuclear medicine doctors. The scores were attributed for both CT and PET images as follows: for images that were illegible the score was 0; for images that were nearly illegible and the contrast was poor the score was 1; for images that were less clear and the contrast was average the score was 2; for images that were clear and had good contrast the score was 3. In addition, the SNR (Signal to Noise Ratio) was obtained from ^18^F-FDG clinical PET images, which equals Av/SD (average value/standard deviation). The same method was adopted for processing tissues from clinical PET/CT images, which were reconstructed without SharpIR.

### Biodistribution

In order to assess the accuracy of the clinical PET/CT imaging quantifications, the mice were immediately sacrificed by cervical dislocation after ^18^F-FDG PET/CT scanning for measuring tumor and organs radioactivity by γ-counter. Tumors and organs including brain, heart, liver, kidney, lung, bone, stomach, small intestine, blood, spleen, skin and muscle were harvested and wet-weighed with a precision scale (± 0.0001 g). The radioactivity of wet tissues was counted for 30s in an automatic γ-counter (WIZARD 2480, Perkin Elmer, New York, USA) [23]. The results of the counts per minute were acquired in Bq and then converted into percentage injected dose per gram (%ID/g) of tissue. The T/NT extraction of the %ID/g from γ-counter was calculated and compared to that from PET images as described above.

### TAC and kinetics of ^18^F-FDG uptake

Dynamic processing software on the Xeleris 2.0 workstation was utilized to manually draw the ROI of blood and tumor on ^18^F-FDG clinical PET images and then to copy the ROI automatically to all the other time frames of that slice. A time-activity curve was extracted from the ROI in each frame [24]. The radioactivity was recorded by averaging all the voxel's values within the ROI. The blood time-activity curve (BTAC) was extracted from the ROI in the heart, and the tumor tissue time-activity curve (TTAC) was derived from the ROI of the highlighted region. To calculate the ^18^F-FDG uptake rate constant K_1_, K_2_, we used the Patlak graphical analysis method for the two reconstructions mentioned above [25]. The arterial plasma input curve acquired from the heart was used as an input function and the tumor tissue time-activity curve was used as an output function for calculating and comparing TACs and K_1_, K_2_ values from different scenarios.

### Statistical analyses

For assessing quantitative accuracy, the correlation between the radioactivity measured by clinical ^18^F-FDG PET/CT in living animals (quantified by %ID/g and SUV_max_) and by γ-counter in tissue samples was tested by linear regression analysis and the Student's t-test. The T/NT values of %ID/g and SUV_max_ calculated from cPET images with SharpIR reconstruction were compared to the T/NT of %ID/g obtained from γ-counter using the Student's t-test. SNR, K_1_, K_2_ values for reconstructions with or without SharpIR were compared using the Student's t-test. The %ID/g both obtained from clinical PET/CT and micro-PET/CT were compared with the Student's t-test. All data were expressed as mean ± SD, *P* value < 0.05 was considered statistically significant.

## Abbreviations

All the abbreviations were embodied in the text.
